# Heterotypic contact inhibition of locomotion can drive cell sorting between epithelial and mesenchymal cell populations

**DOI:** 10.1242/jcs.223974

**Published:** 2019-05-31

**Authors:** Simon Brayford, Fiona N. Kenny, Toru Hiratsuka, Eduardo Serna-Morales, Lawrence Yolland, Andrei Luchici, Brian M. Stramer

**Affiliations:** 1 Randall Centre for Cell and Molecular Biophysics, King's College London, SE1 1UL London, UK; 2 Centre for Stem Cells and Regenerative Medicine, King's College London, SE1 9RT London, UK; 3 Dacian Consulting, 84 Brookwood Road, SW18 5BY London, UK

**Keywords:** Cell sorting, Contact inhibition of locomotion, ERK signalling, MAPK signalling, Ephrin signalling

## Abstract

Interactions between different cell types can induce distinct contact inhibition of locomotion (CIL) responses that are hypothesised to control population-wide behaviours during embryogenesis. However, our understanding of the signals that lead to cell-type specific repulsion and the precise capacity of heterotypic CIL responses to drive emergent behaviours is lacking. Using a new model of heterotypic CIL, we show that fibrosarcoma cells, but not fibroblasts, are actively repelled by epithelial cells in culture. We show that knocking down EphB2 or ERK in fibrosarcoma cells specifically leads to disruption of the repulsion phase of CIL in response to interactions with epithelial cells. We also examine the population-wide effects when these various cell combinations are allowed to interact in culture. Unlike fibroblasts, fibrosarcoma cells completely segregate from epithelial cells and inhibiting their distinct CIL response by knocking down EphB2 or ERK family proteins also disrupts this emergent sorting behaviour. These data suggest that heterotypic CIL responses, in conjunction with processes such as differential adhesion, may aid the sorting of cell populations.

## Abstract (precis)

**Summary:** Active cell repulsion, driven by ephrin and ERK signalling, is required for fibrosarcoma cells to segregate from epithelial cells in culture.

## INTRODUCTION

Contact inhibition of locomotion (CIL) is a process whereby a cell ceases motility or changes trajectory upon collision with another cell. The response can occur between cells of the same type (homotypic) or between different types (heterotypic), leading to distinct dynamics depending on the cell type and the receptors expressed on their surfaces (Stramer and Mayor, 2016).

Our understanding of the functional significance of CIL for animal physiology is only beginning to be elucidated. Differential homotypic and heterotypic CIL dynamics have recently been shown to play a role in animal development (Theveneau et al., 2013) and a loss of heterotypic CIL is hypothesised to be involved in cancer metastasis (Abercrombie, 1979), yet the signals behind these responses are only starting to emerge. In this study, through screening cell types for distinct CIL behaviours, we report that fibrosarcoma cells, rather than losing their heterotypic CIL response after contact with epithelial cells as previously predicted (Abercrombie, 1979), are actively repelled via an Eph–ERK signalling cascade, highlighting that not all cancer cells are necessarily CIL deficient. Interestingly, when fibrosarcoma and epithelial cells are allowed to mix in culture, the heterotypic repulsion between them leads to sorting and segregation of the populations. Recent work has suggested that differential adhesion, the predominant mechanism hypothesised to drive cell sorting, is inadequate in predicting the segregation of mesenchymal populations (Pawlizak et al., 2015; Tambe and Fredberg, 2015) and our data suggest that CIL may be an additional mechanism behind this important developmental phenomenon.

## RESULTS AND DISCUSSION

### Fibroblasts and fibrosarcoma cells exhibit distinct responses upon collision with epithelial cells

**Fig. 1.**
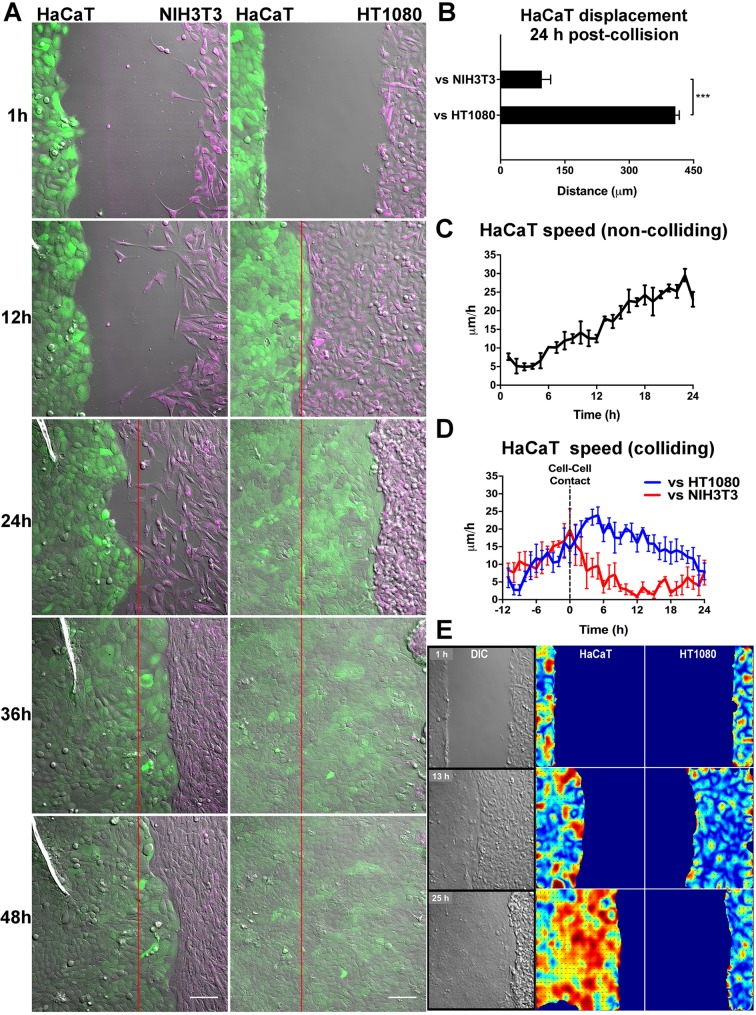
**Fibroblasts and fibrosarcoma cells exhibit distinct responses upon collision with epithelial cells.** (A) Confrontation assay between epithelial cells (HaCaT, green) and fibroblasts (NIH3T3, magenta) or fibrosarcoma cells (HT1080, magenta). Red line indicates collision point. Scale bars: 100 µm. (B) Mean±s.e.m. HaCaT displacement 24 h after colliding with NIH3T3 or HT1080 cells (*n*=3; ***P<0.001, Student's *t*-test). (C) Mean±s.e.m. leading-edge velocity of migrating HaCaT cells when no other cell population is encountered (*n*=3). (D) Mean±s.e.m. leading-edge velocity of migrating HaCaT cells involving collision with either NIH3T3 or HT1080 cell populations. Dotted line indicates collision point (*n*=3). (E) PIV analysis of the HaCaT and HT1080 interaction in A showing global increase in HaCaT velocity despite collision with HT1080 cells. Pseudocolour heatmap from blue to red represents a shift from low to high velocity.

To study heterotypic cell–cell collisions, we used a confrontation assay whereby two different cell populations were allowed to migrate and collide. Following a screen of different epithelial versus mesenchymal cell types, an unexpected phenomenon was revealed. When migrating epithelial cells (HaCaT) encountered migrating fibroblasts (NIH3T3), both populations ceased their forward motion, forming a sharp border (Fig. 1A; Movie 1). In contrast, fibrosarcoma cells (HT1080) seemed to undergo repulsion following collision with epithelial cells (Fig. 1A,B; Movie 1). Interestingly, this repulsive response was not observed with melanoma cell lines (Fig. S1A,B, Movie 2), suggesting that fibrosarcoma cells may have an enhanced heterotypic CIL response.

When no colliding partner was encountered, HaCaT cells migrated with a constantly increasing velocity (Fig. 1C), which was also observed following their collision with fibrosarcoma cells (Fig. 1D), suggesting that epithelial migration was unaffected by collision with this cell type. In contrast, HaCaT migration was severely reduced following collision with fibroblasts (Fig. 1D). The repulsion of fibrosarcoma cells was further analysed using particle image velocimetry, which revealed a population-wide increase in epithelial cell velocity following collision with fibrosarcoma cells (Fig. 1E; Movie 3) showing that fibrosarcoma cells have a distinct response to epithelial cells.

### Fibrosarcoma cells undergo active repulsion from epithelial cells

**Fig. 2.**
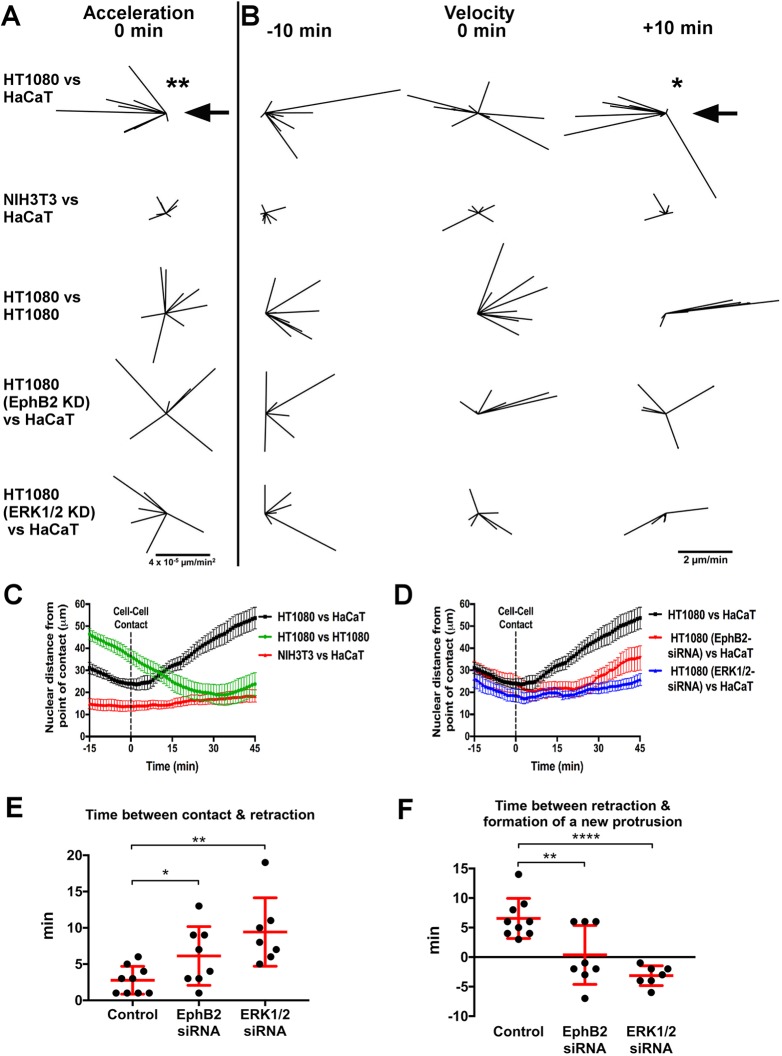
**Fibrosarcoma cells undergo active repulsion upon collision with epithelial cells, which is perturbed by EphB2 or ERK1/2 knockdown.** (A) Vectors depicting acceleration changes upon collision (time=0 min) normalised to the position of the colliding partner (large arrow). A significant rearward acceleration is only observed in fibrosarcoma cell (HT1080) versus epithelial cell (HaCaT) collision (*n*≥6 collisions; **P<0.01, non-parametric Wilcoxon signed-rank test). (B) Vectors depicting velocity of colliding cells before, at time of, and after collision normalised to the position of the colliding partner (large arrow). A significant post-collision rearward velocity away from the colliding partner is only observed in HT1080 versus HaCaT (*n*≥6 collisions; *P<0.05, non-parametric Wilcoxon signed-rank test). (C) Mean±s.e.m. cell distance from colliding partner during heterotypic and homotypic collisions between HT1080 cells, fibroblasts (NIH3T3) and HaCaT cells. Following collision (dotted line), only in the HT1080 and HaCaT combination do cells rapidly separate (*n*≥6 collisions). (D) Mean±s.e.m. cell distance from colliding partner during heterotypic HaCaT–HT1080 collisions comparing non-transfected (HaCaT versus HT1080 from C) with EphB2 or ERK1/2 knockdown HT1080 cells (*n*≥6 collisions). (E) Mean±s.d. time taken for HT1080 cells to retract their leading edge following contact with HaCaT cells (*n*≥7 collisions per condition; *P<0.05, **P<0.01, Student's *t*-test). (F) Mean±s.d. time between leading edge retraction and formation of a protrusion in HT1080 cells following contact with HaCaT cells. Negative values indicate that formation of the new protrusion occurred before leading edge retraction (*n*≥7 collisions; **P<0.01, ****P<0.0001, Student's *t*-test).

To determine whether fibrosarcoma cell repulsion by epithelial cells was an active process, we examined individual cell collisions (Movie 4). Analysis of acceleration changes before and after collisions revealed that when fibrosarcoma cells collided with epithelial cells there was a rapid, rearward acceleration (Fig. 2A) suggesting a sudden change in motion. This response was similar to previously reported collisions between *Drosophila* macrophages, which also undergo a classical CIL response involving active repulsion (Davis et al., 2012; Davis et al., 2015). The backward acceleration of fibrosarcoma cells was accompanied by a shift in the direction of their velocities before, during and after the collision as fibrosarcoma cells were repelled from epithelial cells (Fig. 2B). In contrast, repulsion was not observed when fibroblasts collided with epithelial cells, nor during homotypic fibrosarcoma collisions, where cells continued to migrate toward the colliding partner after collision (Fig. 2A,B). Plotting the distance from collision over time revealed that heterotypic collisions led to fibrosarcoma cells slowing before migrating away from epithelial cells, in contrast to homotypic collisions, which led to their continued forward motion (Fig. 2C). These data highlight that fibrosarcoma cells show distinct CIL dynamics involving active repulsion in response to collision with epithelial cells.

### ERK activation downstream of EphB2 induces heterotypic repulsion of fibrosarcoma cells

There is much evidence for ephrin (Eph) receptors playing a role in epithelial cell repulsion and cell segregation (Porazinski et al., 2016; Poliakov et al., 2008). However, less is known about whether Eph signalling may control the repulsion and segregation of mesenchymal cell populations. HT1080 fibrosarcoma cells express the ephrin receptor EphB2 (Fig. S2A), knockdown of which (Fig. S2A) abolished the backward acceleration upon collision with epithelial cells (Fig. 2A). This led to a random distribution of cell velocities after collision (Fig. 2B), suggesting that cells were randomly migrating away from epithelial cells rather than being actively repelled. Similarly, plotting the distance from collision over time revealed that EphB2 knockdown in fibrosarcoma cells slowed their separation from epithelial cells, further showing that the repulsion phase of CIL was disrupted (Fig. 2D). We also tested the effect of knocking down EphB2 in the confrontation assay and found that indeed, collective repulsion of fibrosarcoma cells following collision with a monolayer of epithelial cells was also impaired (Fig. S2C,D).

One signalling pathway previously reported to be controlled by Eph receptor activation is the extracellular signal-regulated kinase (ERK) pathway. However, there is conflicting evidence for ERK signalling specifically downstream of Eph receptor activation, with some reports suggesting inhibition (Miao et al., 2003; Elowe et al., 2001) and others reporting an increase following Eph receptor stimulation (Pratt and Kinch, 2002; Vindis et al., 2003; Kandouz et al., 2010). We found that knockdown of ERK1 and ERK2 (also known as MAPK3 and MAPK1, respectively) in fibrosarcoma cells (Fig. S2B) phenocopied EphB2 knockdown both in single-cell collisions (Fig. 2A,B,D) and in confrontation assays (Fig. S2C,D). To confirm that these effects were related to collisions and not general migration defects, we examined non-colliding fibrosarcoma cells and found no significant difference in either velocity (Fig. S2E) or persistence (Fig. S2F) following EphB2 or ERK knockdown.

We also examined the temporal relationship between protrusion and retraction events surrounding collisions. We found that both EphB2 and ERK1/2 knockdown in fibrosarcoma cells led to a delay in leading edge retraction following contact with epithelial cells (Fig. 2E). This was accompanied by a disruption to the protrusion-retraction sequence where, unlike control cells that retracted their leading edge before forming a new protrusion, both EphB2 and ERK1/2 knockdown cells often formed a new protrusion prior to leading edge retraction (Fig. 2F), suggesting a loss of coordinated retraction and repolarisation of lamellae after collision. Interestingly, ERK proteins were recently shown to regulate various cytoskeletal and migratory behaviours (Yang et al., 2018; Mendoza et al., 2011; Aoki et al., 2017) and we hypothesise that the ERK family is a good candidate to modulate distinct CIL dynamics.

**Fig. 3.**
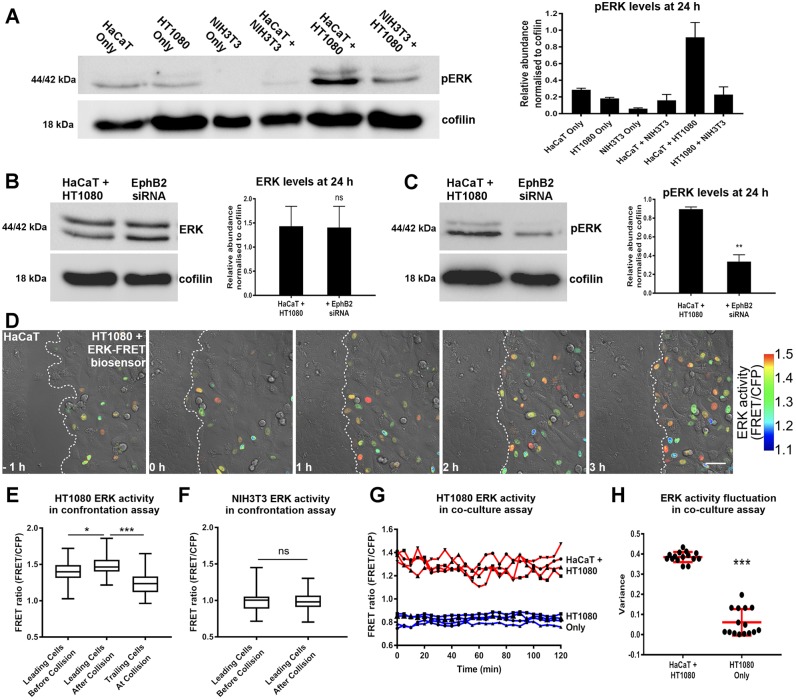
**ERK activation is elevated in collisions between epithelial and fibrosarcoma cells.** (A) Western blot and quantification of mean±s.e.m. band intensity reveals pERK was highest during co-culture of epithelial (HaCaT) and fibrosarcoma (HT1080) cells (*n*=3). (B,C) Western blot and quantification of mean±s.e.m. band intensity showing that total ERK was unaffected (B) but pERK was significantly reduced (C) by EphB2 knockdown in HT1080 cells co-cultured with HaCaT cells, compared with non-transfected control (*n*=3; **P<0.01; ns, not statistically significant; Student's *t*-test). All western blot quantification data in A–C normalised to expression of cofilin. (D) Confrontation assay between HaCaT cells (unlabelled) and HT1080 cells expressing the ERK FRET biosensor. Scale bar: 50 µm. (E) ERK activity in the leading row of HT1080 cells in the confrontation assay 30 min before collision, 30 min after collision and internal control (trailing cells at collision) (*n*=29 cells per condition, boxplots represent range, median and quartiles; *P<0.05, ***P<0.001, Mann–Whitney test). (F) ERK activity in the leading row of fibroblasts (NIH3T3) in the confrontation assay before and after collision (*n*=23 cells per condition, boxplots represent range, median and quartiles; ns, not statistically significant; Mann–Whitney test). (G) ERK activity time course of four representative HT1080 cells cultured alone (blue) or co-cultured with HaCaT cells (red). (H) Mean±s.d. fluctuation of ERK activity as measured by variance in randomly selected tracks of HT1080 cells cultured alone or co-cultured with HaCaT cells (*n*=15 tracks; ***P<0.001, Mann–Whitney test).

To examine ERK activity during heterotypic interactions, we used western blotting to probe lysates from co-cultures for phosphorylated ERK proteins (pERK) and found that pERK was elevated among epithelial–fibrosarcoma co-cultures compared with either of these cell types individually (Fig. 3A), suggesting that ERK activity increased when these cell types interacted. We also found that EphB2 knockdown in fibrosarcoma cells, whilst not affecting total ERK levels (Fig. 3B), resulted in a significant decrease in pERK in epithelial–fibrosarcoma co-cultures (Fig. 3C), suggesting that ERK activation is downstream of EphB2 in the fibrosarcoma population.

To analyse ERK activation dynamics during individual cell–cell interactions, we used a FRET biosensor that has previously been demonstrated to report ERK activity in living cells (Komatsu et al., 2011). Confrontation assays using cells expressing the ERK biosensor revealed that fibrosarcoma cells increased ERK activity following collision with HaCaT cells (Fig. 3D,E; Movie 5). In contrast, fibroblasts did not increase ERK activity upon collision with HaCaT cells (Fig. 3F; Fig. S3A), which is consistent with our western blotting results of co-cultures (Fig. 3A).

We also examined co-cultures of fibrosarcoma and epithelial cells, and found higher ERK activity among fibrosarcoma cells in the presence of epithelial cells compared to fibrosarcoma cells alone (Fig. 3G). Finally, by measuring variance in ERK activity compared to fibrosarcoma only, we found that cells in the mixed group had a higher fluctuation in their ERK activity over time (Fig. 3H), and we hypothesise that this was due to fibrosarcoma cells constantly undergoing heterotypic collisions throughout the course of the assay. Taken together, these data suggest that ERK signalling acts downstream of EphB2 and is involved in the repulsion of fibrosarcoma cells upon their contact with epithelial cells.

### Fibrosarcoma and epithelial cell populations sort in culture through CIL interactions

**Fig. 4.**
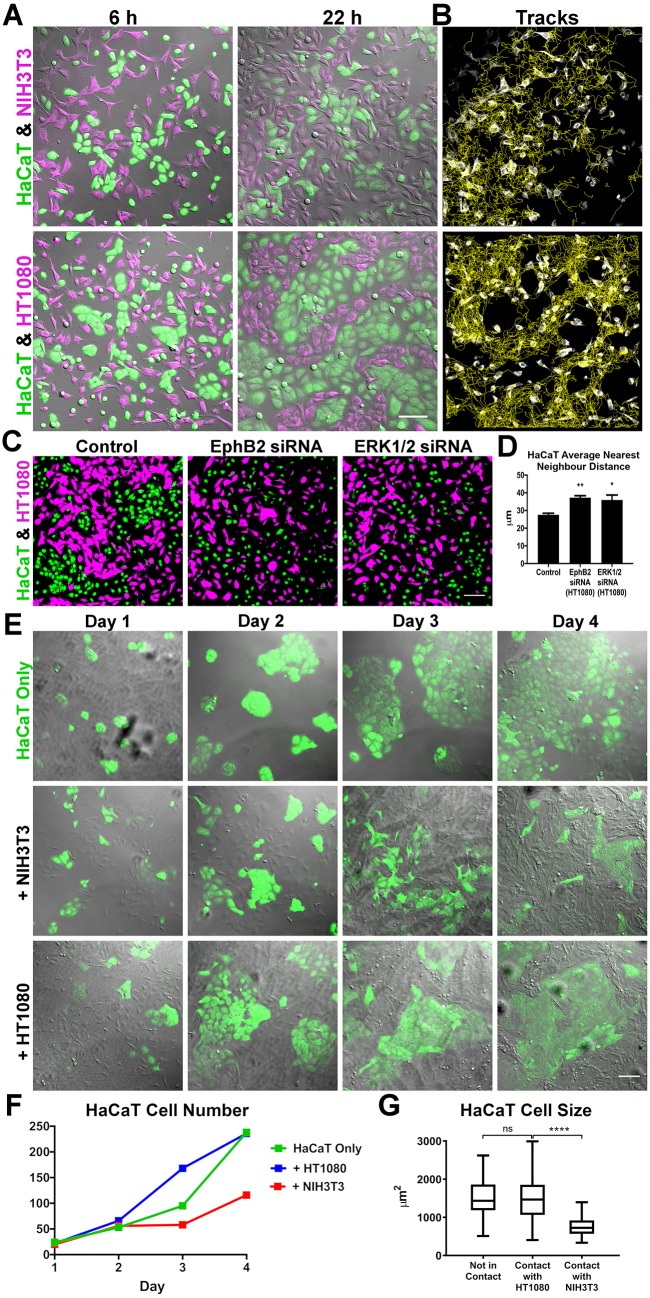
**Fibrosarcoma cells sort from epithelial cells in culture, a behaviour which is disrupted by EphB2 or ERK1/2 knockdown.** (A) Snapshots (from Movie 6) of co-cultured epithelial cells (HaCaT, green) with fibroblasts (NIH3T3, magenta) or with fibrosarcoma cells (HT1080, magenta). Scale bar: 100 µm. (B) Tracks of NIH3T3 or HT1080 cells as shown in A and taken throughout Movie 6. (C) Images (fixed after 24 h) of HaCaT cells (green) co-cultured with control, or EphB2 or ERK1/2 knockdown HT1080 cells (magenta). Scale bar: 100 µm. (D) Mean±s.e.m. dispersion of HaCaT cells in C quantified by measuring their distribution of nearest neighbour distances (*n*=3; **P<0.01, *P<0.05, Student's *t*-test). (E) Long-term culture of HaCaT cells (green) alone or co-cultured with NIH3T3 or HT1080 cells (unlabelled). Scale bar: 100 µm. (F) HaCaT cell number when grown in isolation or co-cultured, showing reduced HaCaT proliferation when co-cultured with NIH3T3 cells but not HT1080 cells. (G) Size of individual HaCaT cells showing reduced spreading when in contact with NIH3T3 cells but not HT1080 cells (*n*≥69 cells per condition, boxplots represent range, median and quartiles; ****P<0.0001; ns, not statistically significant; Mann–Whitney test,).

To examine how heterotypic CIL affects population dynamics, we generated time-lapse movies of co-cultures, and found that epithelial cells and fibroblasts formed a homogeneous, interspersed population. In contrast, epithelial and fibrosarcoma cells immediately began to sort from one another (Fig. 4A; Movie 6). In addition, automated tracking of the mesenchymal cells in these movies revealed a streaming-type behaviour specifically among fibrosarcoma cells (Fig. 4B; Movie 6), highlighting this population's coordinated segregation from epithelial cells over time.

To examine whether the specific repulsive CIL dynamics of fibrosarcoma cells in response to epithelial cells was driving their segregation, we inhibited Eph or ERK signalling in the fibrosarcoma population. Indeed, knockdown of EphB2 or ERK1/2 in fibrosarcoma cells resulted in a disruption to their segregation from epithelial cells (Fig. 4C,D). Similarly, treatment with U0126, an inhibitor of ERK signalling, also resulted in disruption to the segregation between fibrosarcoma and epithelial cells (Fig. S3B–D). These data suggest that segregation of fibrosarcoma from epithelial cells is driven by active repulsion between these two populations.

We also investigated the phenomenon of cell sorting over a longer period to examine the final patterns of the populations (Fig. 4E). Alone, epithelial cells grew in clusters that eventually merged to form large colonies. However, the presence of fibroblasts resulted in much smaller clusters as the two cell populations reached equilibrium. In contrast, when grown together with fibrosarcoma cells, which proliferate at a similar rate to fibroblasts (Fig. S3E), epithelial cells formed large colonies unimpeded by the presence of fibrosarcoma cells (Fig. 4E). Furthermore, epithelial cell proliferation was specifically reduced in the presence of fibroblasts (Fig. 4F), suggesting they may undergo contact inhibition of proliferation in the absence of a repulsive heterotypic CIL response. Finally, we measured the size of individual epithelial cells and found that those in contact with fibroblasts failed to spread compared with those in contact with fibrosarcoma cells (Fig. 4G; Fig. S3F). These data suggest that the repulsion of fibrosarcoma cells allows the epithelial cells to spread, which may enable their proliferation, similar to other epithelial cell types (Gumbiner and Kim, 2014; Aragona et al., 2013).

The differential adhesion hypothesis (DAH) states that two different cell types can segregate simply by taking into account their differential adhesion and surface tension (Foty and Steinberg, 2013). This has been the predominant model to explain cell sorting and assumes that cells segregate in a liquid-like phase separation. The problem with this concept is that groups of cells do not behave like perfect liquids; cell clusters can quickly go from a fluid-like to a solid-like state as cell density increases in a process termed ‘jamming’, which leads to cells rapidly slowing their motion (Sadati et al., 2013). Furthermore, the DAH has recently been found inadequate in predicting the sorting of mesenchymal cells (Pawlizak et al., 2015; Tambe and Fredberg, 2015).

Here, we show that a mesenchymal cell type can rapidly segregate from an epithelial population. Differential adhesion is still playing a role in this case. The high surface tension of the epithelial population and the near-non-existent mutual adhesion of the fibrosarcoma cells, leads to the less cohesive cell type (HT1080) surrounding the more cohesive cell type (HaCaT), as predicted by DAH (Foty and Steinberg, 2013). However, DAH is insufficient to explain the observed results on its own, as inhibiting heterotypic repulsion prevents sorting. We hypothesise that CIL between fibrosarcoma and epithelial cells helps fluidise the population, allowing cells to sample their adhesive landscape. Furthermore, there appears to be an aspect of fibrosarcoma CIL relieving contact inhibition of proliferation of epithelial cells, allowing epithelial colonies to grow and merge (Fig. S4). It is therefore possible that these results also highlight an unexplored connection between CIL and contact inhibition of proliferation despite these processes being thought of as distinct behaviours (Stramer and Mayor, 2016).

It was previously hypothesised that one hallmark of cancer cells is a loss of CIL in response to contact with neighbouring cells, which may aid their metastatic spread. However, here we reveal that not all cancer cells are deficient in heterotypic CIL; indeed, fibrosarcoma cells show a robust repulsive CIL response after contact with epithelial cells. One question is whether such a response is physiologically relevant. It is possible that both positive and negative CIL behaviours of some cancer cells observed *in vitro* may be related to an ontogenetic theory of cancer dissemination. It was recently hypothesised that some cancers may spread through permissive compartments that are defined embryologically (Höckel, 2012; Höckel, 2015), and it will be interesting to determine whether differential CIL dynamics may be playing a role in the compartmentalised spreading of metastatic cells *in vivo*.

## MATERIALS AND METHODS

### Cell culture

Cell lines were a gift from Vicky Sanz-Moreno and Matthias Krause (Randall Centre for Cell and Molecular Biophysics, King's College London, UK) and were routinely tested and found to be free from mycoplasma contamination. Cells were maintained in DMEM (6429; Sigma-Aldrich) supplemented with 10% FBS, at 37°C and 5% CO_2_. For routine maintenance, cells were cultured in T75 plastic cell culture flasks and split via trypsinisation approximately every 3 days or when approaching confluence.

### Cell labelling

CellTracker Green CMFDA and CellTracker Red CMTPX dyes (Invitrogen) were used to differentially label cell types in confrontation and co-culture assays. Cells were exposed to either dye at 1 µM in serum-free medium for 30 min at 37°C before being washed once with PBS, trypsinised and re-suspended in complete medium.

### Imaging

Images were acquired using an LSM 880 inverted confocal microscope (Zeiss). Cells were maintained at 37°C and 5% CO_2_ for the duration of live imaging. Images were acquired using differential interference contrast (DIC) imaging along with airyscan filter sets for 488 or 561 lasers with either a 20× (NA 0.45) air objective or 40× (NA 0.95) oil objective (Zeiss).

### Confrontation assay

Two-well culture inserts (Ibidi) were placed into the centre of the well of a 24 well µ-Plate (Ibidi). Two different pre-labelled cell types were seeded into opposite chambers at a density of 1×10^5^ cells/cm^2^ and incubated overnight. The culture insert was then removed and the well topped up with 1 ml culture medium before live imaging of the resulting 500 µm gap for ∼48 h at 20× magnification.

### Particle image velocimetry (PIV)

Time-lapse images were manually segmented prior to analysis and pseudo-speckle analysis was performed as described previously (Betz et al., 2009). The size of the search image was chosen such that it spanned the maximum expected displacement of the cells during the acquisition time. To cover the whole search image, a cross-correlation coefficient was computed between source image and a sub-image of the search image shifted by one pixel. Finally, a spatial convolution with a Gaussian kernel and temporal convolution were used to interpolate the measured displacements to cover all the pixels within the frame. The complete algorithm for this analysis, including the filtering and interpolation was implemented in MATLAB (MathWorks).

### Individual cell collisions and manual tracking

Analysis of single-cell collisions was carried out by combining cells pre-labelled with CellTracker dyes (Invitrogen) at low density, plating sparsely and allowing cells to adhere for 4–6 h before imaging at 40× magnification. For the kinematics analysis, a collision was defined as the time point at which the cell in question contacts any part of the colliding partner. The cell nucleus, which could be identified from DIC movies, was manually tracked using the mTrackJ plugin for ImageJ (NIH) to calculate the *x*,*y* coordinates of the cell at all time points.

### Kinematics analysis

Kinematics analysis of the velocity and acceleration of cells was calculated as previously described (Dunn and Paddock, 1982; Davis et al., 2015). In order to assess the statistical significance of the direction of cells after collision, a binomial test with a probability of success of 95% was performed on the cell velocity unit vectors every five minutes from 5 min before to 20 min after collision. To assess the statistical significance of acceleration, a one-sample *t*-test of the horizontal component of the vectors was performed.

### Gene silencing by small interfering RNA (siRNA)

HT1080 cells were plated onto 6-well plates at 2×10^5^ cells/well and allowed to attach overnight. Cells were transfected with pre-validated siRNA sequences to knockdown human EphB2 (EHU060511; Sigma-Aldrich) or human ERK1/2 (6560; Cell Signaling Technology). siRNA was transfected using Lipofectamine RNAiMAX reagent (Invitrogen) according to the manufacturer's instructions. Experiments were carried out 48 h post-transfection.

### Western blotting and antibodies

Total cellular proteins from individual cells or co-cultured populations were prepared by rinsing cells with cold PBS and scraping with RIPA buffer [20 mM Tris pH 7.4, 150 mM sodium chloride, 1% (v/v) Nonidet P-40, 0.5% (w/v) sodium deoxycholate, 1 mM EDTA, 0.1% (w/v) SDS] in the presence of protease and phosphatase inhibitor cocktails (Roche Diagnostics). 20 μg of protein per well was resolved on SDS-PAGE gels before electro-transfer to PVDF membranes. Following blocking in 5% (w/v) BSA in Tris-buffered saline (TBST), immunoblotting was performed using anti-EphB2 (83029; Cell Signaling Technology), anti-GADPH (ABS16; Millipore), anti-ERK1/2 (9102; Cell Signaling Technology), anti-cofilin (5175; Cell Signaling Technology) or anti-phospho-ERK1/2 (Thr202/Tyr204) (9101; Cell Signaling Technology) antibodies at 1:1000 dilution. Membranes were then washed with TBST and incubated with species-appropriate HRP-conjugated IgG secondary antibodies (Dako, Agilent Technologies) at 1:10,000 dilution. Chemiluminescence was measured using ImageJ after applying Clarity western ECL substrate (BioRad).

### FRET biosensor transduction

The lentiviral plasmid of nucleus-localised FRET biosensor for ERK (EKAREV-NLS) has been previously characterised (Komatsu et al., 2011) and was a kind gift from Michiyuki Matsuda at Kyoto University, Japan. EKAREV-NLS ERK biosensor was expressed in HT1080 cells by lentiviral transduction. EKAREV-ELS in replication-defective, self-inactivating lentiviral pCSII vector was co-transfected with packaging plasmid (pCAG-HIVgp) and VSV-G-/Rev-expressing plasmid (pCMV-VSVG-RSV-Rev) into Lenti-X 293T cells (Clontech). High-titre viral solution was prepared and used for transduction into cells. NIH3T3 cells were transfected with a pPBbsr-EKAREV-NLS plasmid encoding EKAREV-NLS in pPB piggyback backbone using jetPRIME reagent (Polyplus-transfection) according to manufacturer's instructions.

### FRET imaging

FRET images were obtained following methods previously reported (Aoki and Matsuda, 2009). Cells were imaged with an LSM 880 inverted confocal microscope (Zeiss) using a 40× (NA 0.95) oil objective (Zeiss). Donor fluorescence protein was excited with a 458 nm laser to obtain donor and FRET signal by detection filters 483/32 and 542/27, respectively. ERK activity was determined by ratiometry (FRET/CFP) as validated previously (Aoki and Matsuda, 2009; Komatsu et al., 2011). Pseudocolour images of ERK activity were shown in intensity modulated display (IMD).

### Short-term co-culture assays

Cells were pre-labelled with either CellTracker Red or Green, counted and combined in 1:1 suspension so that 5×10^4^ of each cell type was seeded per well in a 24-well imaging µ-Plate (Ibidi) for a total of 1×10^5^ cells/well. For wild-type assays, cells were allowed 6 h to adhere before being imaged overnight for a total of 24 h. For RNAi experiments, cells were transfected with siRNA for 48 h before being differentially labelled, combined, and allowed to mix for 24 h before fixation with 4% paraformaldehyde in PBS. All nuclei were then labelled with DRAQ5 (Invitrogen) before coverslips were mounted. Images were then acquired, and the green channel was used to create a mask over the nuclei of HT1080 cells, leaving only the nuclei of HaCaT cells visible for segmentation. Nearest neighbour distances were then calculated in ImageJ. For U0126 MAPK/ERK kinase (MEK) inhibition movies, cells were differentially labelled before being combined and allowed to adhere for 6 h. U0126 (Sigma-Aldrich) was then added at a concentration of 20 µM to inhibit MEK/ERK signalling alongside DMSO as vehicle control, and live imaging commenced for a total of 24 h. For quantitative analysis, HaCaT cells at 6 h and 24 h were manually segmented and nearest neighbour distances were calculated in ImageJ.

### Automated cell tracking

For automated tracking, the image channel corresponding to NIH3T3 or HT1080 cells was thresholded in ImageJ so that individual cells could be detected as particles in the TrackMate plugin in order to generate cell tracks.

### Long-term co-culture assay

HaCaT cells were pelleted by centrifugation and re-suspended in CellTrace CFSE (Invitrogen) at a working concentration of 5 µM in PBS and incubated for 20 min at 37°C. Cells were again pelleted and re-suspended in fresh culture medium to a density of 2×10^4^ cells/ml and incubated for 10 min to allow the CFSE reagent to undergo acetate hydrolysis. This suspension was then combined with either DMEM (control), an NIH3T3 cell suspension (unlabelled) or HT1080 cell suspension (unlabelled) and added to coverslips in 24-well plates at a total of 20,000 cells/well and incubated for 4 days, fixing with 4% paraformaldehyde every 24 h and mounting coverslips with ProlongGold (Invitrogen). Coverslips were then imaged at 20× magnification for further analysis using ImageJ.

### Proliferation assay

NIH3T3 and HT1080 cells were seeded in a 96-well plate at 1000 cells/well and incubated for 4 h. Growth medium was then carefully removed and 1× CyQUANT NF (Invitrogen) dye reagent was added. Cells were then incubated at 37°C for 1 h. This incubation period is required for equilibration of dye–DNA binding, resulting in a stable fluorescence endpoint. The fluorescence intensity of each sample was measured every 24 h using a fluorescence microplate reader with excitation at ∼485 nm and emission detection at ∼530 nm. Fresh growth medium was added every 48 h. Cell numbers at each time point were determined using a standard curve as per the manufacturer's instructions.

### Statistical analyses

Statistical analyses are described in each figure legend.

## Supplementary Material

Supplementary information
