# Supplementary material for: Heterotypic contact inhibition of locomotion can drive cell sorting between epithelial and mesenchymal cell populations
Source: J Cell Sci. 2019 May 31;132(11):jcs223974. doi: 10.1242/jcs.223974 (PMC6589087; doi:10.1242/jcs.223974)
Supplement: Supplementary information [file joces-132-223974-s1.pdf]

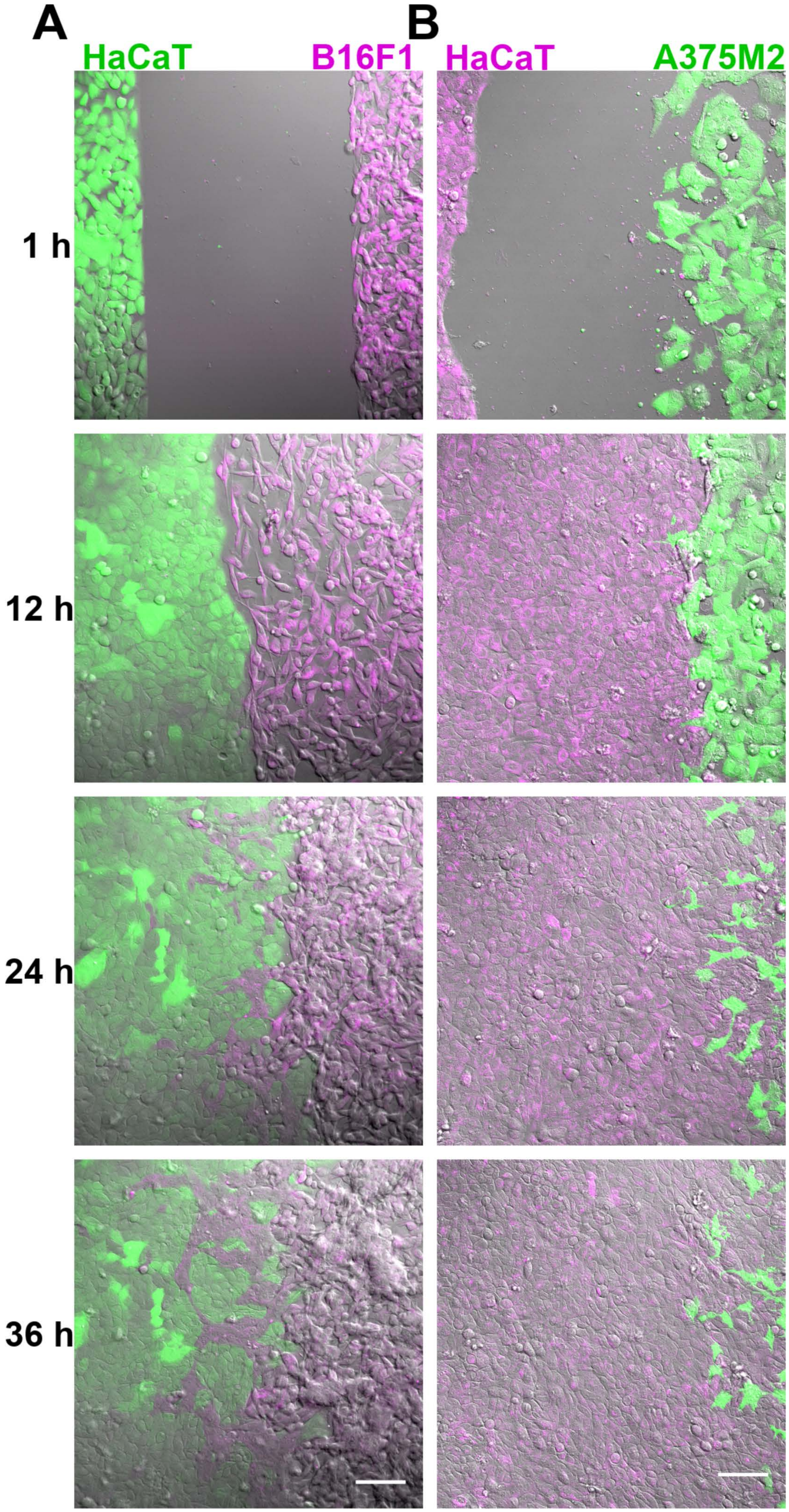
A
HaCaT
B16F1
B
HaCaT
A375M2
1 h
12 h
24 h
36 h

Figure S1.

(A) Confrontation assay between human epithelial cells (HaCaT, green) mouse melanoma cells (B16F1, magenta).

(B) Confrontation assay between human epithelial cells (HaCaT, magenta) and human melanoma cells (A375M2, green).

Scale bars = 100 µm.

J. Cell Sci.: doi:10.1242/jcs.223974: Supplementary information

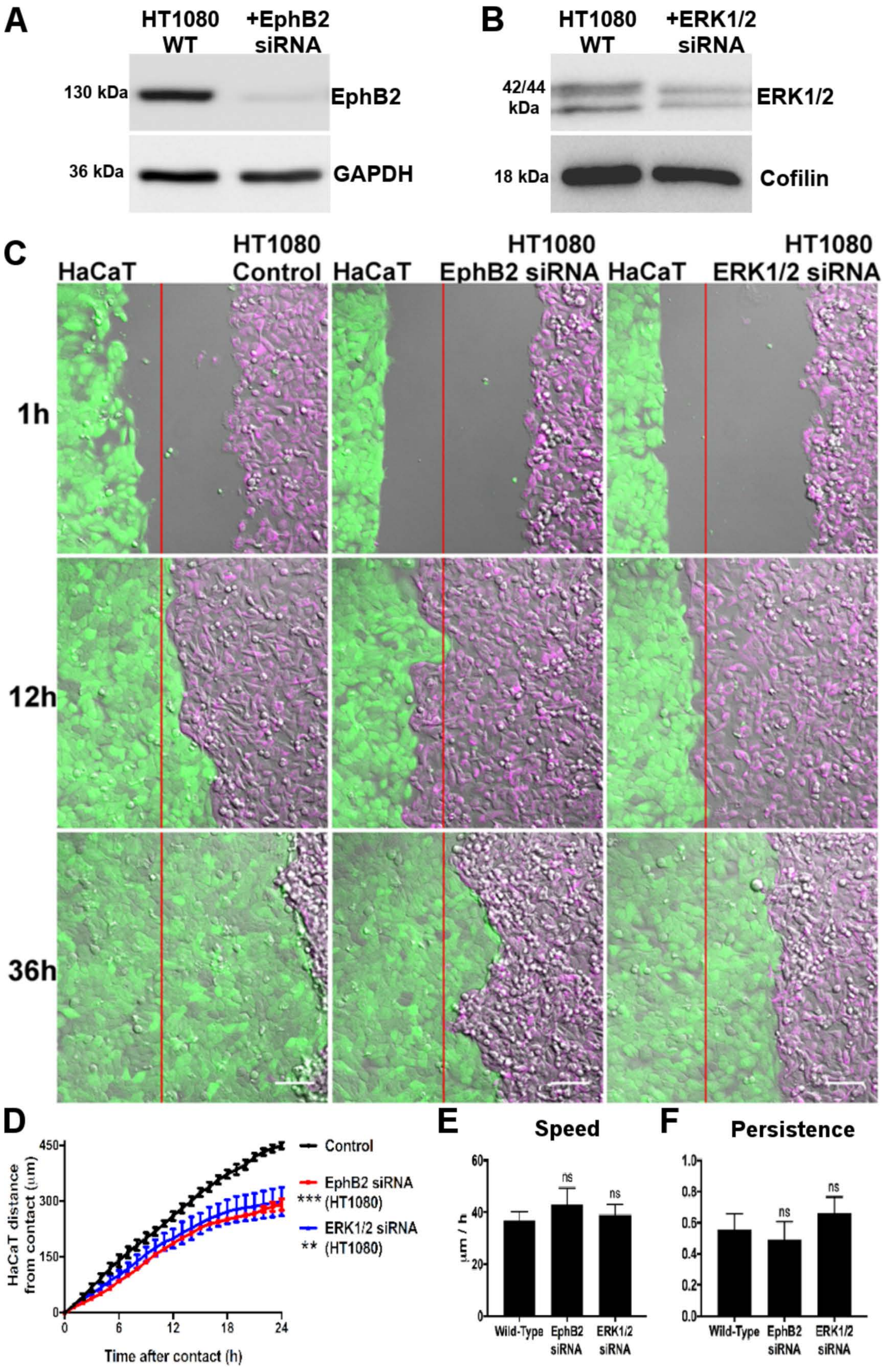

Figure S2.

(A) Western blot confirming knockdown of EphB2 protein in HT1080 cells 48 h post-transfection with siRNA compared with non-transfected cells (WT = Wild Type).

(B) Western blot confirming knockdown of ERK1/2 protein in HT1080 cells 48 h post-transfection with siRNA compared with non-transfected cells (WT = Wild Type).

(C) Screenshots from a confrontation assay in which HaCaT cells (green) are allowed to collide with either Control, EphB2 or ERK1/2 siRNA transfected HT1080 cells (magenta). Red line indicates position of contact. Scale bars = 100 µm.

(D) Displacement of HaCaT leading-edge after collision with HT1080 cells (Control, EphB2 or ERK1/2 siRNA) in the confrontation assay. (n = 3, error bars = SEM, ***P < 0.001, **P < 0.01, Friedman test).

(E) Speed of non-colliding HT1080 cells transfected with either EphB2 or ERK1/2 siRNA compared with non-transfected (wild-type). (n = 10 cells, error bars = SEM, ns = not statistically significant, Student's t-test).

(F) Persistence of non-colliding HT1080 cells transfected with either EphB2 or ERK1/2 siRNA compared with non-transfected (wild-type). (n = 10 cells, error bars = SEM, ns = not statistically significant, Student's t-test).

Journal of Cell Science • Supplementary information

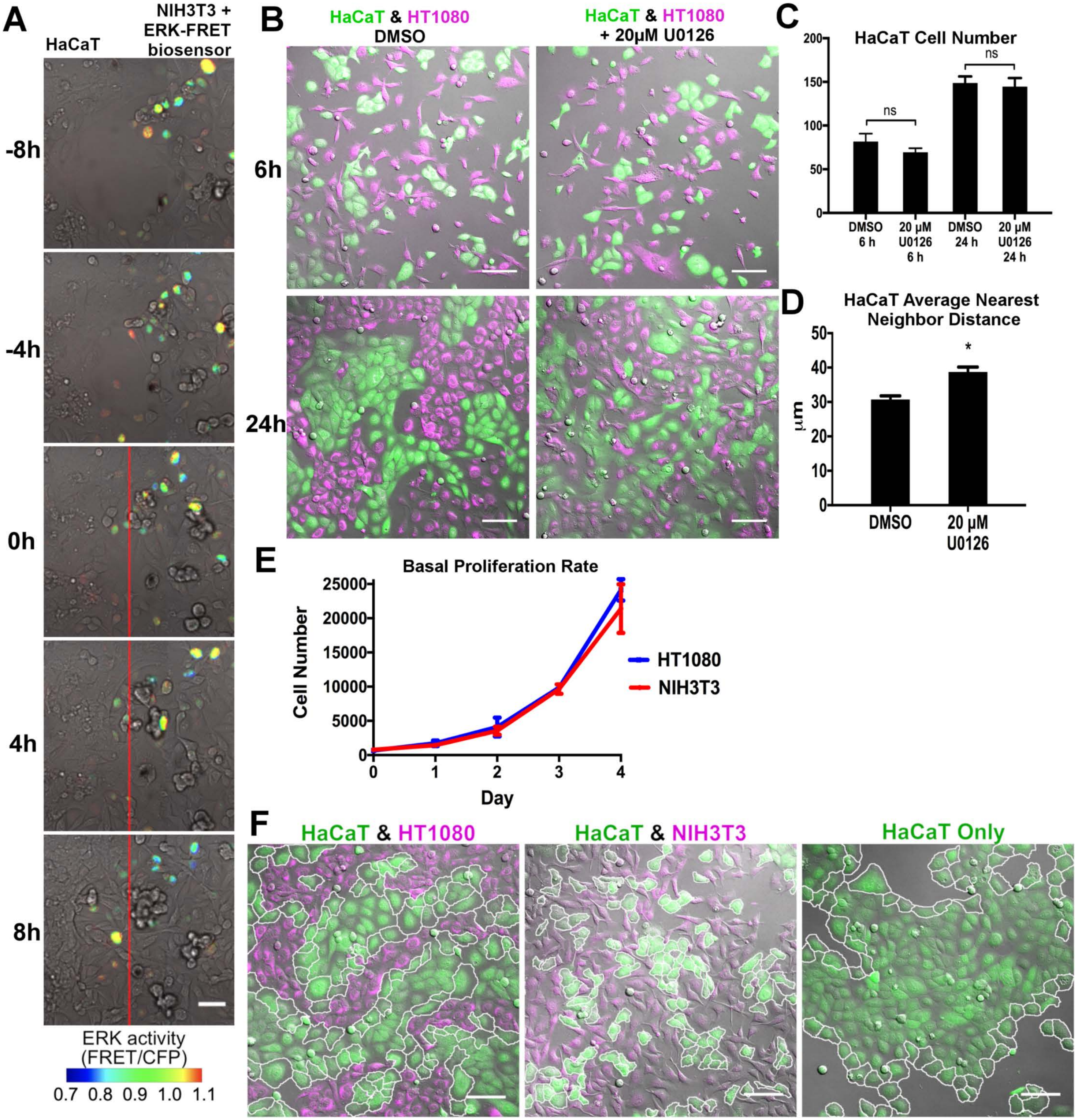
A
HaCaT
NIH3T3 + ERK-FRET biosensor
-8h
-4h
0h
4h
8h
ERK activity (FRET/CFP)
0.7 0.8 0.9 1.0 1.1
B
HaCaT & HT1080 DMSO
HaCaT & HT1080 + 20μM U0126
6h
24h
C
HaCaT Cell Number
ns
DMSO 6 h
20 μM U0126 6 h
DMSO 24 h
20 μM U0126 24 h
D
HaCaT Average Nearest Neighbor Distance
μm
DMSO
20 μM U0126
E
Basal Proliferation Rate
Cell Number
Day
HT1080
NIH3T3
F
HaCaT & HT1080
HaCaT & NIH3T3
HaCaT Only

Figure S3.

(A) Confrontation assay between HaCaT cells (unlabelled) and NIH3T3 cells expressing the ERK FRET-biosensor. Scale bar = 100 µm.
(B) Images of co-cultures of epithelial cells (HaCaT, green) with fibrosarcoma cells (HT1080, magenta) comparing DMSO vehicle control with 20 µM U0126 treatment to inhibit ERK signalling.
(C) Quantification of HaCaT cell numbers at 6 and 24 h to demonstrate that U0126 does not selectively impact the growth rate of HaCaT cells. (n = 3, error bars = SEM, ns = not statistically significant, Student's t-test).
(D) The dispersion of HaCaT cells at 24 h quantified by measuring their distribution of nearest neighbor distances. An increase in HaCaT dispersion represents a reduction in their segregation from HT1080 cells. (n = 3, error bars = SEM, *P < 0.05, Student's t-test).
(E) Basal proliferation rates of NIH3T3 and HT1080 cells (n = 3, error bars = mean ± SD).
(F) Screenshots showing the extent of spreading (quantified in Fig. 4G) of epithelial cells (outlined in white) when contacting HT1080 cells (left), NIH3T3 cells (middle), and when not in contact with either mesenchymal cell population (cultured alone, right).

Journal of Cell Science • Supplementary information

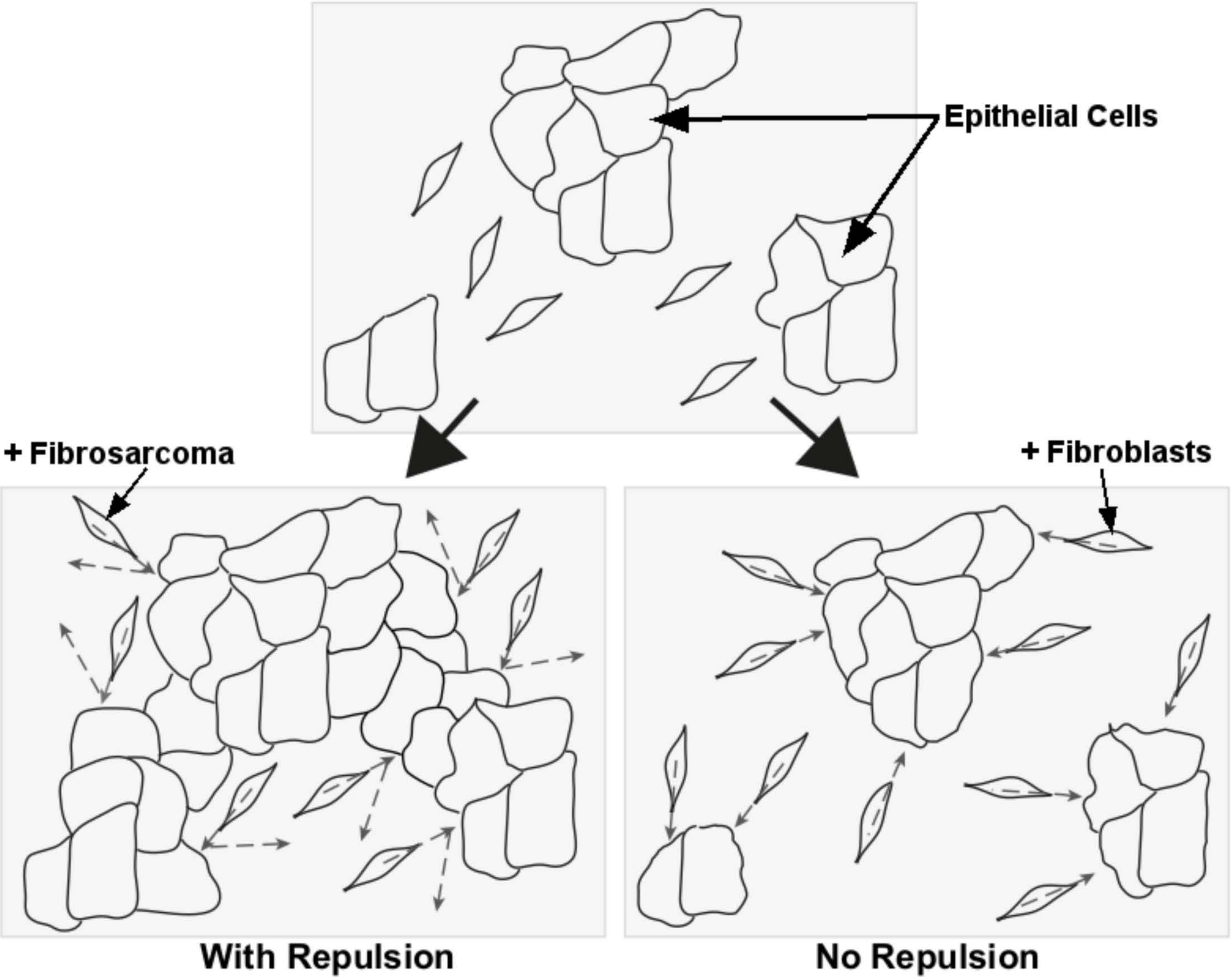

Figure S4. Schematic depicting a role for CIL in cell sorting between a mesenchymal cell-type and an epithelial population. In the presence of a repulsive interaction (left panel) the epithelial cells are given space to spread and subsequently proliferate, which allows the epithelial colonies to grow and merge, and ultimately sort from the mesenchymal cells. In the absence of repulsion (right panel) the epithelial cells fail to spread due to spatial constraints, which leads to a reduction in their proliferation. In this case the lack of repulsive CIL prevents the epithelial colonies from expanding and merging with neighbouring colonies, which prevents the sorting process.

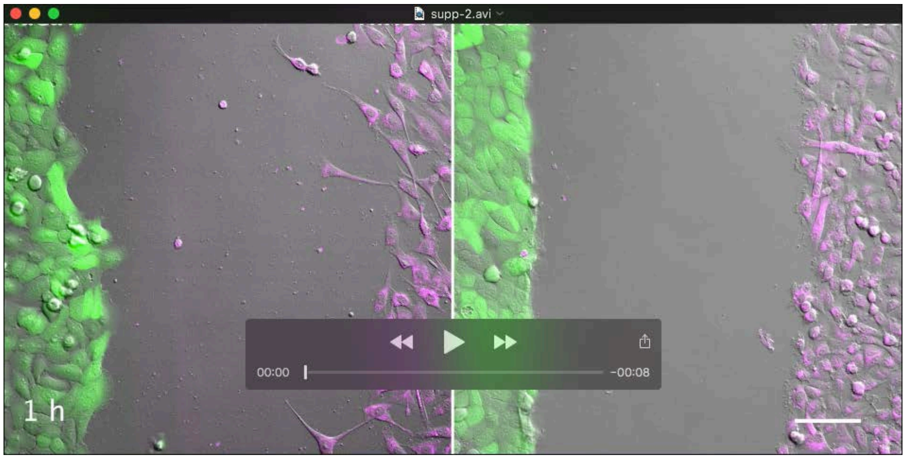

Movie 1.
Confrontation assays between HaCaT epithelial cells (green) and NIH3T3 fibroblasts or HT1080 fibrosarcoma cells (magenta). 1h frames. Scale bar = 100 μm.

Journal of Cell Science • Supplementary information

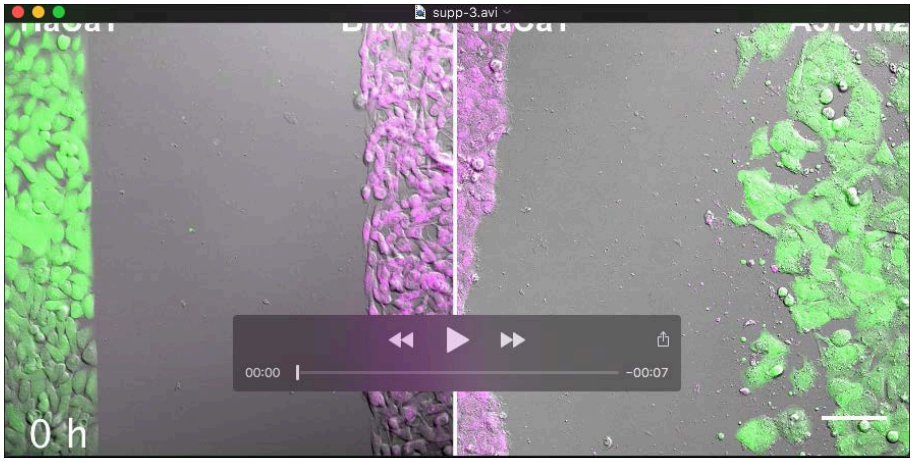

Movie 2.

Confrontation assays between:

(Left) Human epithelial cells (HaCaT, green) and mouse melanoma cells (B16F1, magenta).

(Right) Human epithelial cells (HaCaT, magenta) and human melanoma cells (A375M2, green).

1 h frames. Scale bar = 100 µm.

Journal of Cell Science • Supplementary information

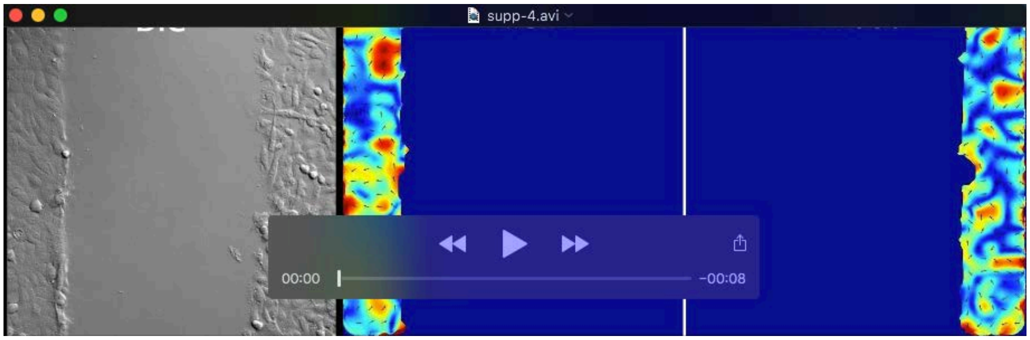

Movie 3.

Particle Image Velocimetry (PIV) heat-map of the HaCaT and HT1080 interaction showing global increase in HaCaT cell speed after colliding with HT1080 cells. Blue to red represents a shift from low to high instantaneous velocity.
1 h frames.

Journal of Cell Science • Supplementary information

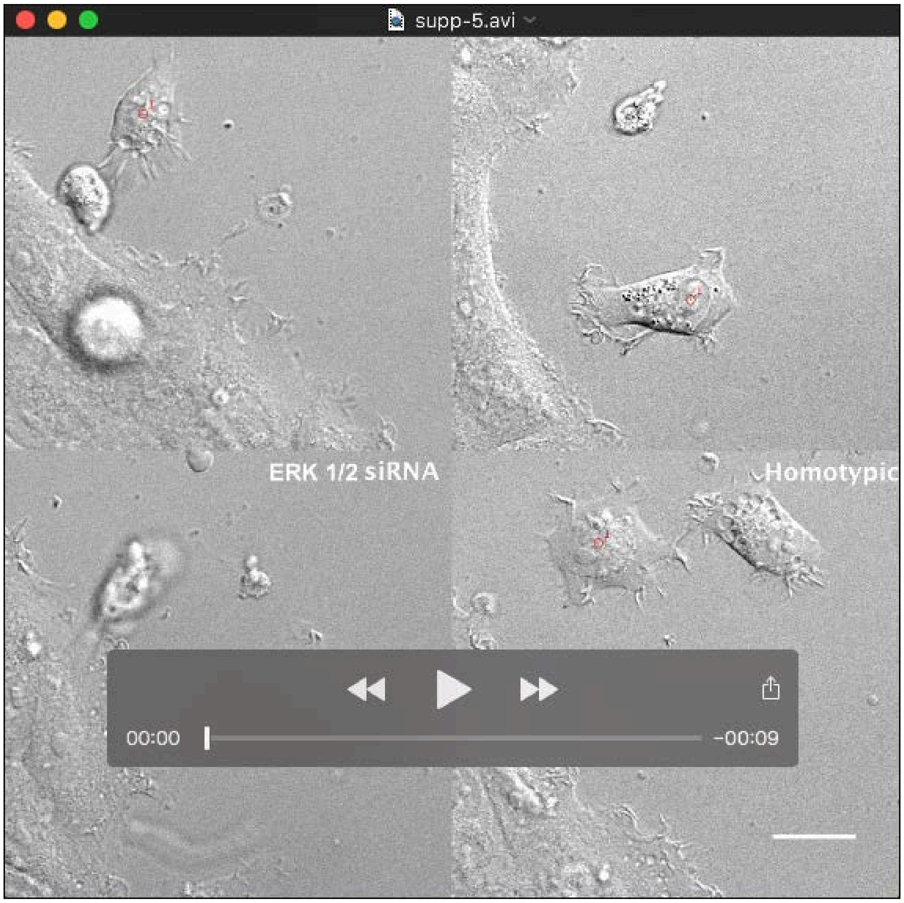

Movie 4.
Individual HT1080 fibrosarcoma cells (Control, EphB2 or ERK1/2 siRNA transfected) colliding with HaCaT epithelial cells or homotypic collisions between HT1080 cells. Red lines represent manual tracking used for the kinematic analysis. 10 sec frames. Scale Bar = 20 μm.

Journal of Cell Science • Supplementary information

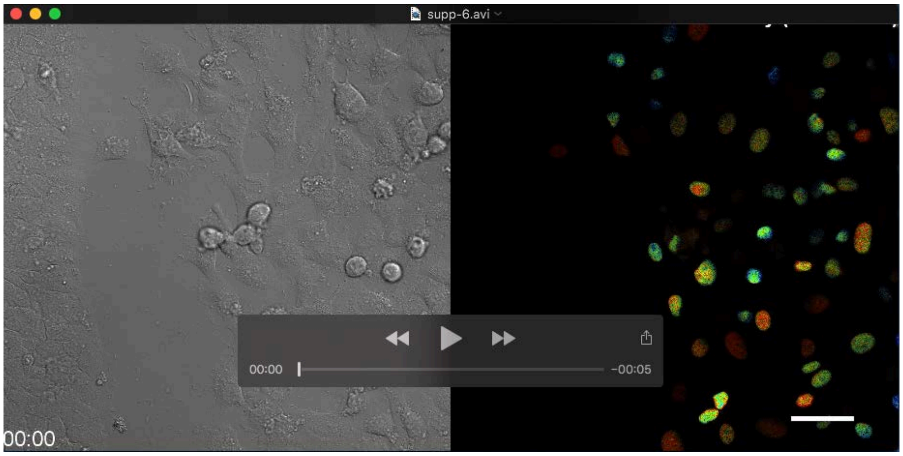

Movie 5.

Confrontation assay between HaCaT cells (unlabelled) and HT1080 cells expressing the ERK FRET-biosensor. Blue to red represents an increase in ERK activity (FRET/CFP).
5 min frames. Scale bar = 50 µm.

J. Cell Sci.: doi:10.1242/jcs.223974: Supplementary information

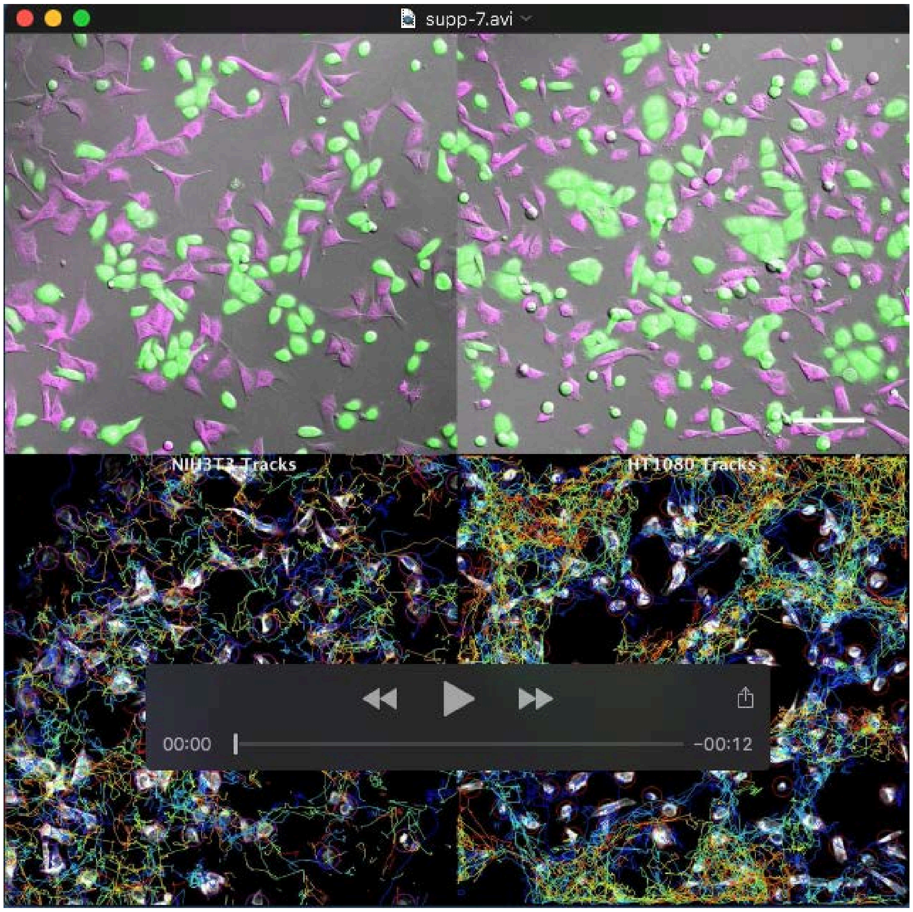

Movie 6.

(Top) Co-culture assay whereby HaCaT cells (green) are co-cultured with either NIH3T3 cells (left, magenta) or HT1080 cells (right, magenta). 10 min frames. Scale bar = 100 μm. (Bottom) Tracks of NIH3T3 fibroblasts (left) and HT1080 fibrosarcoma cells (right) from the movies above. Tracks shift blue to red throughout the movie to demonstrate that, in the HT1080 case (bottom right), cells become segregated from HaCaT cells (not labelled) over time.
